# Brompton Consumption Hospital

**Published:** 1902-06-07

**Authors:** 


### BROMPTON CONSUMPTION HOSPITAL.

PLACIDITY enlivened by hopefulness was, as is usually the case, the prevailing note of the sixty-first annual court of governors of the Hospital for Consumption, Brompton, held in the handsome boardroom of the institution on Thursday, 29th ult. Only nine governors were present, and of these all but one—a lady—were members of the Committee of Management; a fact which that body naturally takes to be an indication of the general feeling that affairs are in good hands.

The chair was taken by Major-General Eaton, and after the minutes of the committee meetings had been read by Mr. Theobald, the secretary, and confirmed, the adoption of the report for the year was moved by the Duke of Wellington. From this it appeared that the total receipts were £37,869, of which £18,794 were from legacies; and the total expenditure £32,728. The in-patients numbered 1,565, of whom 1,134 were discharged, many greatly benefited; 179 died; and there remained in the hospital 252. The average stay of each patient was 73 days, and the daily average number of beds occupied was 257. In the out-patients' department there were 12,830 new cases, with 66,490 attendances. Satisfaction was expressed that the King had become an annual subscriber of 10 guineas. Chief prominence was given to the fact that progress was being made with the scheme for a country branch and convalescent home where the open-air treatment can be applied. The site had been acquired at Heatherside, near Frimley, in Surrey. Out of the £55,000 required, only £5,400 had so far been received or promised, leaving some £50,000 to complete the cost of what was expected would be the best "open-air" hospital in the world, capable of accommodating 100 patients. Among the contributions already received were £1,000, and 100 guineas from the Goldsmiths' and the Mercers' Companies respectively, and £550 from the Council of King Edward's Fund.

One other matter only the chairman touched upon in his brief address—namely, that they had unexpectedly been put to considerable expense during the year by the discovery that all their ceilings were in so dilapidated a condition that their repair had to be taken in hand at once.

After the adoption of the report the Committee of Management was re-elected; and the following noblemen were elected vice-presidents—namely, the Duke of Portland, the Marquis of Zetland, Field-Marshal Earl Roberts, Viscount Portman, and Lord Rothschild.

Thanks to the medical staff and to the chairman concluded the meeting, the cordial applause with which the latter vote was carried being a distinct relief after the calm—not to say dulness—which had till then enveloped the proceedings.

